# Navigational instinct: a reason not to live trap deer mice in residences.

**DOI:** 10.3201/eid0501.990125

**Published:** 1999

**Authors:** C H Calisher, W P Sweeney, J J Root, B J Beaty


### Navigational Instinct: A Reason Not to Live Trap Deer Mice in Residences

**To the Editor:** Although the rodent that most often invades homes in North America is the house mouse, *Mus musculus*, the deer mouse, *Peromyscus maniculatus*, principal vertebrate host of Sin Nombre virus (SNV) (1), also invades homes (2), particularly in rural areas. Barring deer mice from human habitations would prevent domiciliary acquisition of SNV. Current recommendations (3) are to prevent wild rodents from entering homes or to snap trap (kill) them should they enter.

To conduct longitudinal studies of hantaviruses in southeastern Colorado on a former cattle ranch now returning to its natural condition as short-grass prairie, we often stay in an old bunkhouse, used by many research groups at irregular intervals. The house, furnished with beds and full kitchen facilities, is well maintained but has openings through which mice can pass to and from the outside. For safety and cleanliness, we removed mice we found inside the house, but between April 1996 and April 1998, we live trapped and released them rather than snap trapping them. Before release the rodents were identified to species; were measured and assessed regarding general appearance and health, sexual preparedness, and presence of wounds; were bled for antibody tests; and were ear-tagged. Nineteen deer mice and one pinyon mouse (a *P. truei*, which did not return) were examined and tagged. At first, we simply released these animals approximately 50 m from the house, but when we realized that they were returning, we released them at increasing distances (50 m to 1,500 m) from the house; the distances were measured by pace counts by at least two investigators.

Three deer mice had been captured multiple times in our test grid (as far as 250 m from the house) before they were first captured in the house. Once captured in the house, however, they were not captured in traps of the grid (i.e., outside the house). The mean distance traversed by the five deer mice that returned to the house was at least 394 m; one mouse returned after being released 500 m and 1,000 m, then 750 m, and 1,200 m from the house at consecutive daily trapping sessions of 3 days. Sometime within the subsequent 6 weeks, this mouse returned to the house from the 1,000-m release point and then from 750 m and 1,200 m away on consecutive days within our 3-day trapping period. Each of the mice returning to the house did so within 24 hours of release, two as few as 6 hours after release from 500 m and 750 m away. Nine mice were captured once; six of eight mice captured twice were captured at least once more; one was captured 10 times, one 7 times, one 6 times, one 4 times, and two 3 times. Equal numbers of male and female, adult and juvenile mice were captured in the house, but only adult mice (5 of 5) returned to the house. Returning deer mice maintained or gained weight between captures and grew in length at approximately the same rate as deer mice captured in the test grid.

Some rodents have been documented to move similar distances (e.g., 1,200 m), but they took more than 2 weeks to complete the trek (4). Homing ability, site fidelity, and navigational proficiency of rodents are well documented (5,6). Teferi and Millar (7) studied the homing ability of deer mice in Alberta, Canada; 50% of deer mice in that study returned to their home sites (a short-grass prairie habitat). The mice traveled 650 m to 1,980 m (mean 1,500 m) and had to cross a river and pass optimal habitat patches to reach their home sites. Deer mice with previous homing experience were more successful in returning home (100%) than inexperienced mice (60%) and faster in doing so (8). Teferi and Millar (7) suggest that these deer mice were able to navigate in a direct route to their home sites. We released mice in locations where they had no direct route to the house; they had to follow a winding road, climb over rocky outcroppings nearly 17 m high, or otherwise surmount obstacles and dangers, such as predators (7).

None of the mice we captured had immunoglobulin G (IgG) antibody to SNV. However, infected deer mice released and then returning to a house or uninfected deer mice released, infected, and then returning to a house would increase the likelihood of human contact with an SNV-infected mouse. The risk would be the same for other hantaviruses infecting other peridomestic rodents. Against current recommendations that rodents in homes be snap trapped, some homeowners live trap and release them outside their homes. Our data strongly support snap trapping mice in homes and provide evidence that released wild mice return and may place the residents at risk.

### Acknowledgments

We thank T. Davis, S.B. Calisher, and E. Kuhn for their assistance in completing these studies.

This work was partially funded by contract U50-CCU-813420-01 from the U.S. Centers for Disease Control and Prevention.

**Charles H. Calisher,* William P. Sweeney,† J. Jeffrey Root,* and Barry J. Beaty***
*Colorado State University, Fort Collins, Colorado, USA; and †University of Texas Medical Branch, Galveston, Texas, USA

### References

1. Childs JE, Ksiazek TG, Spiropoulou CF, Krebs JW, Morzunov S, Maupin GO, et al. Serologic and genetic identification of *Peromyscus maniculatus* as the primary rodent reservoir for a new hantavirus in the southwestern United States. J Infect Dis 1994;169:1271-80.
2. Glass GE, Johnson JS, Hodenbach GA, DiSalvo LJ, Peters CJ, Childs JE, et al. Experimental evaluation of rodent exclusion methods to reduce hantavirus transmission to humans in rural housing. Am J Trop Med Hyg 1997;56:359-64.
3. Centers for Disease Control and Prevention. Hantavirus infection—southwestern United States: interim recommendations for risk reduction. MMWR Morb Mortal Wkly Rep 1993;42(RR-11):1-13.
4. Ostfeld RS, Manson RH. Long-distance homing in meadow voles, (*Microtus pennsylvanicus*). Journal of Mammalogy 1996;77:870-3.
5. August PV, Ayvazian SG, Anderson JGT. Magnetic orientation in a small mammal, *Peromyscus leucopus*. Journal of Mammalogy 1989;70:1-9.
6. Fluharty SL, Taylor DH, Barrett GW. Sun compass orientation in the meadow vole, *Microtus pennsylvanicus*. Journal of Mammalogy 1976;57:1-9.
7. Teferi T, Millar JS. Long distance homing by the deer mouse, *Peromyscus maniculatus*. Canadian Field-Naturalist 1993;107:109-11.
8. Robinson WL, Falls JB. A study of homing of meadow mice. American Midland Naturalist 1965;73:188-224.

### *Bartonella quintana* in Body Lice Collected from Homeless Persons in Russia

**To the Editor:** Lice are obligate blood-feeding insects; three lice species (*Pediculus humanus* var *capitatis*, *P. humanus* var *corporis*, and *Phtirus pubis*) have been connected with humans throughout history. The body louse (*P. humanus corporis*) is the vector for three infectious diseases: epidemic typhus caused by *R. prowazekii*, trench fever caused by *B. quintana*, and relapsing fever caused by *Borrelia recurrentis* (1-3). Infestation with the body louse is associated with cold weather, poverty, and poor hygiene. In Russia, louse-transmitted diseases have caused more deaths than any other infectious disease in recent centuries (4). During the last decade, pediculosis (infestation with *P. humanus*) has increased markedly throughout the world (5,6), especially in developing countries and in areas (e.g., Eastern Europe, Russia) that have undergone vast social and economic changes. The incidence of pediculosis in Russia is approximately 220 to 300 cases per 100,000 inhabitants (7). Social and economic upheavals in the former Soviet Union have increased the number of homeless people, among whom pediculosis is highly prevalent (6).

A disease of the past, epidemic typhus, has reemerged as a public health concern after a 1996 outbreak in Burundi, the largest outbreak of the disease since World War II (5,8). During World War II, a huge typhus epidemic caused illness in more than 20,000,000 people in Russia. *R. prowazekii* infection can persist in a latent form in convalescent typhus patients, remanifesting itself in a recrudescent form (Brill-Zinsser disease) in patients under stress (1). Sporadic cases of Brill-Zinsser disease are reported every year in all regions of the former Soviet Union (9) and because most of the population has no immunity to *R. prowazekii,* the risk for a typhus outbreak is increased. In a recent outbreak in the Lipetsk region, 360 km from Moscow, 24 louse-infested, febrile patients in an unheated psychiatric institution had serologically diagnosed typhus (10).

The great epidemics of trench fever in Europe took place during World War I (2). However, recently a large outbreak of trench fever associated with epidemic typhus has been reported in Burundi (5). Sporadic cases of *B. quintana* infection have occurred during the last decade in Europe and the United States, mainly in HIV-infected patients, the homeless, and persons with chronic alcoholism; the infection has manifested itself as trench fever, bacteremia, bacillary angiomatosis, or endocarditis (11-16). Relapsing fever has not been reported in Russia for more than 50 years, despite a high prevalence after the 1917 revolution and during World War II (17).

We studied the presence of typhus, trench fever, and relapsing fever agents in body lice

